# Evaluation of left ventricular diastolic function: state of the art after 35 years with Doppler assessment

**DOI:** 10.1007/s12574-017-0364-2

**Published:** 2017-12-13

**Authors:** Otto A. Smiseth

**Affiliations:** Division of Cardiovascular and Pulmonary Diseases, Department of Cardiology and Institute for Surgical Research, Center for Cardiological Innovation and Center for Heart Failure Research, Oslo University Hospital and University of Oslo, Rikshospitalet, 0027 Oslo, Norway

**Keywords:** Left ventricular diastolic function, Heart failure with preserved ejection fraction, Pulmonary venous velocities, Mitral velocities, Left atrial pressure, Left ventricular filling pressure

## Abstract

Left ventricular (LV) diastolic function can be evaluated by echocardiographic indices of LV relaxation/restoring forces, diastolic compliance, and filling pressure. By using a combination of indices, diastolic function can be graded and LV filling pressure estimated with high feasibility and good accuracy. Evaluation of diastolic function is of particular importance in patients with unexplained exertional dyspnea or other symptoms or signs of heart failure which cannot be attributed to impaired LV systolic function and to assess filling pressure in patients with heart failure and reduced LV ejection fraction. Furthermore, grading of diastolic dysfunction can be used for risk assessment in asymptomatic subjects and in patients with heart disease.

## Introduction

Left ventricular systolic function is evaluated clinically by measuring left ventricular (LV) ejection fraction (EF) and more recently by strain imaging as a supplementary method. Evaluation of LV diastolic function is more challenging, and a number of different noninvasive approaches have been proposed. Recently, important consensus was reached regarding use of echocardiography to assess diastolic function [1], and two large multicenter studies using invasive pressure measurement as reference method confirmed the validity of the new recommendations as a means to diagnose heart failure with preserved ejection fraction (HFpEF) [2, 3]. However, appropriate use and interpretation of echocardiographic indices of diastolic function require understanding of the physiology of LV filling. The present article reviews key elements of this physiology and how echocardiography can be used to diagnose diastolic dysfunction and identify elevated LV filling pressure in patients with suspected HFpEF.

**Fig. 1:**
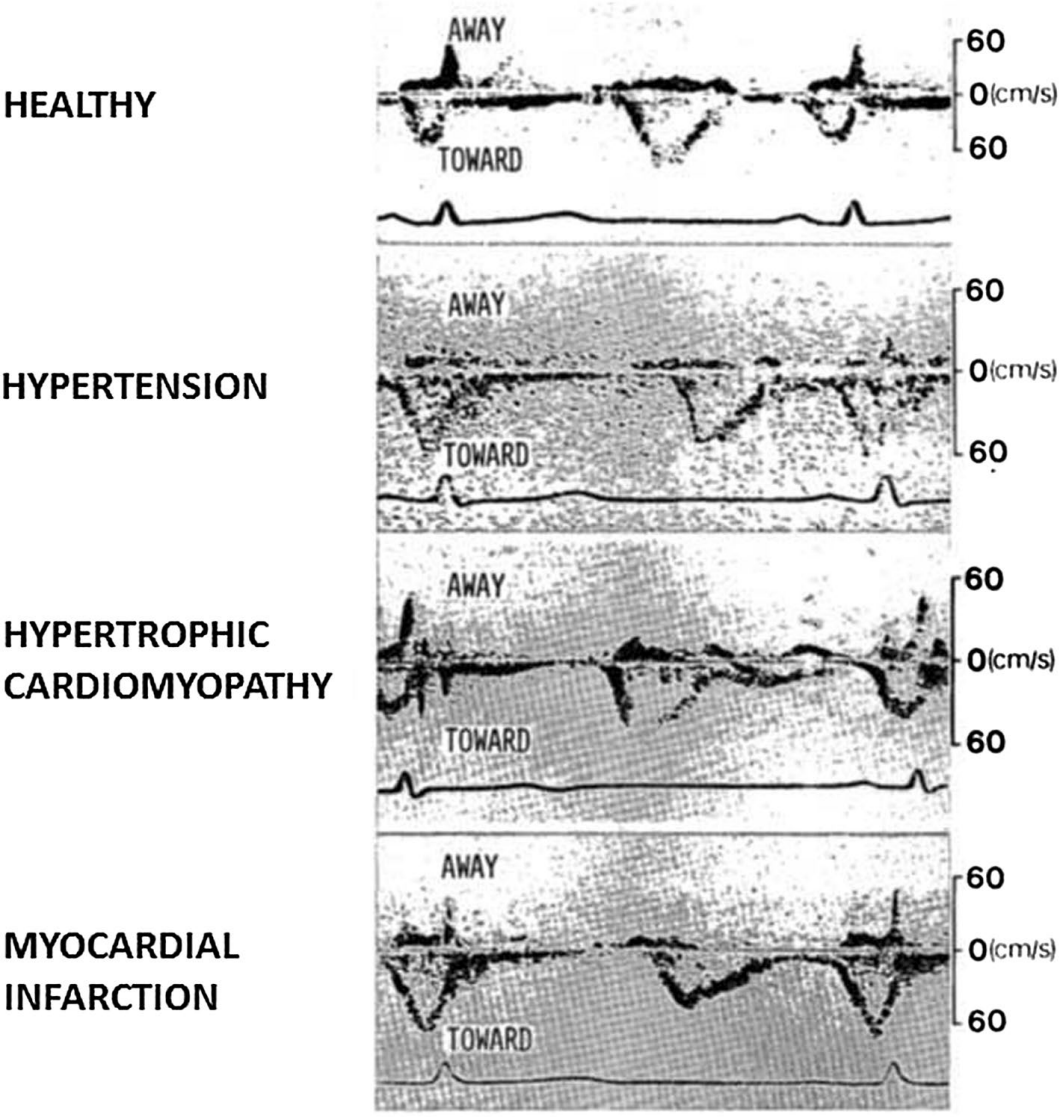
Recordings by Kitabatake et al. [4] of transmitral blood flow velocities by pulsed Doppler echocardiography in a healthy individual and in three patients: In the healthy individual the highest velocity is in early diastole, whereas the patients with hypertension and myocardial infarction have highest velocity in late diastole. Each of the three patients have reduced deceleration rate of early mitral velocity. Note that the polarity of velocities is opposite to current standards

To my knowledge, the first study of LV diastolic function using transthoracic Doppler echocardiography was published 35 years ago by Kitabatake et al. [4, 5] at Osaka University Medical School, who applied pulsed Doppler to measure mitral flow velocities. They showed that the normal transmitral filling pattern with dominant early velocity (*E*) and smaller atrial-induced velocity (*A*) was disturbed in arterial hypertension and in specific heart diseases. In hypertension and in myocardial infarction, there was *E*/*A* reversal and reduced deceleration rate of early filling (Fig. 1). This study has stood the test of time, as these filling patterns are still used as main diagnostic criteria for diastolic dysfunction.

## Introduction to diastolic function

**Fig. 2:**
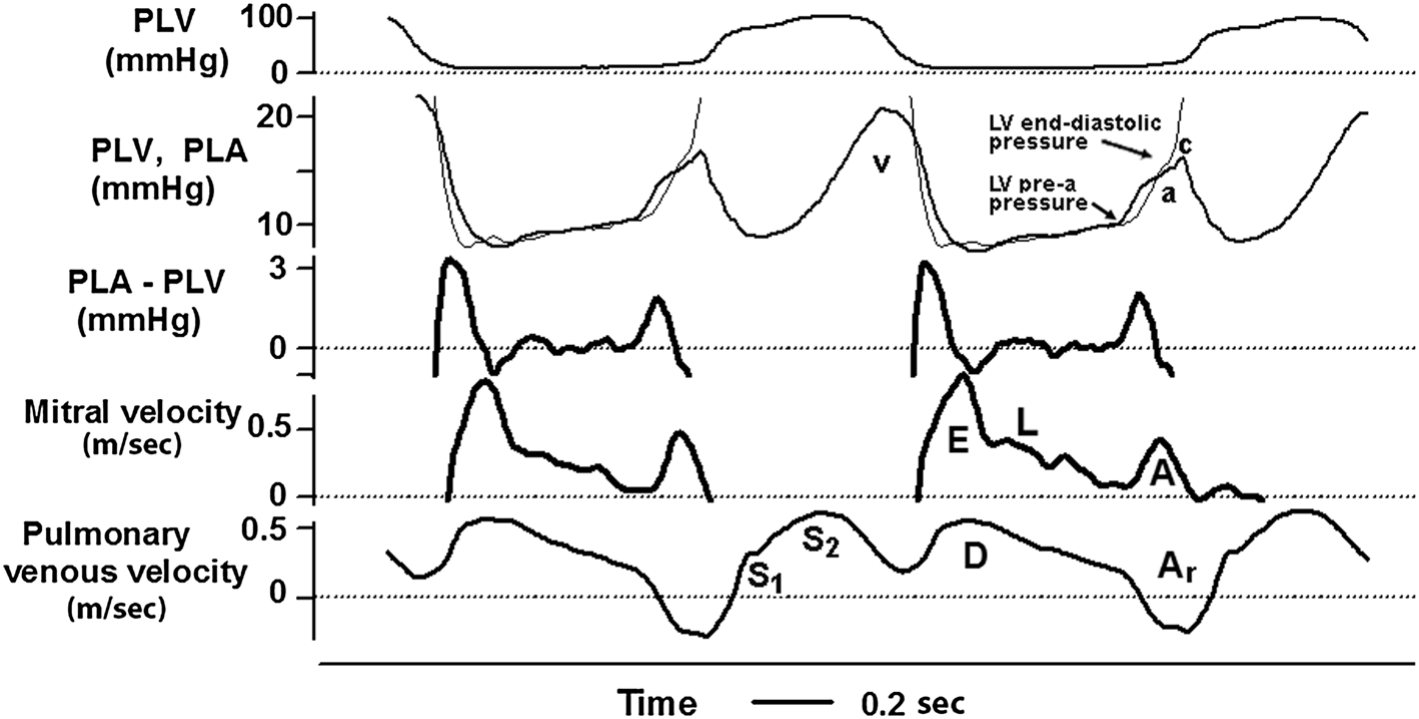
Intraoperative recordings in a patient with coronary artery disease. Left ventricular (PLV) and left atrial (PLV) pressures were measured by micromanometer, mitral velocity by electromagnetic sensor, and pulmonary venous velocity by ultrasound transit time. PLA–PLV is the transmitral pressure gradient. “a” and “v” indicate LA pressure waves. *E*, *A*, and *L* are transmitral early, atrial-induced, and mid-diastolic velocities, and *S*_1_, *S*_2_, *D*, and *A*_r_ are pulmonary venous early systolic, late systolic, diastolic, and atrial-induced reversed velocities, respectively. Modified from Smiseth and Thompson [27]

During isovolumic relaxation, LV pressure falls rapidly, and when it has declined below atrial pressure, a pressure gradient is established between the atrium and the ventricle, the mitral valve opens, and the ventricle fills rapidly, giving rise to the *E*-velocity (Fig. 2). During diastasis, left atrial (LA) and LV pressures almost equilibrate and transmitral flow occurs at a low rate. Not infrequently in patients with diastolic dysfunction, there may be a velocity peak during mid-diastole (*L*-velocity). Finally, atrial contraction causes late diastolic filling, giving rise to the *A*-velocity.

The transmitral pressure gradient represents the driving force for transmitral flow and, therefore, as illustrated in Fig. 2, mitral velocity increases as long as the pressure gradient is positive, and peak velocity occurs when the pressure gradient is zero. Then the gradient reverses, and the negative gradient represents the force which decelerates mitral flow. The etiology of the *L*-velocity is not entirely clear, but it is associated with elevated LV diastolic pressure and impaired myocardial relaxation [1]. As seen in Fig. 2, after the sharp *E* deceleration, mitral flow continues during diastasis with no measurable pressure gradient. Velocity with no pressure gradient may represent inertia of *E* and possibly in part momentum-driven flow due to blood entering the LA from the pulmonary veins during diastasis.

**Fig. 3:**
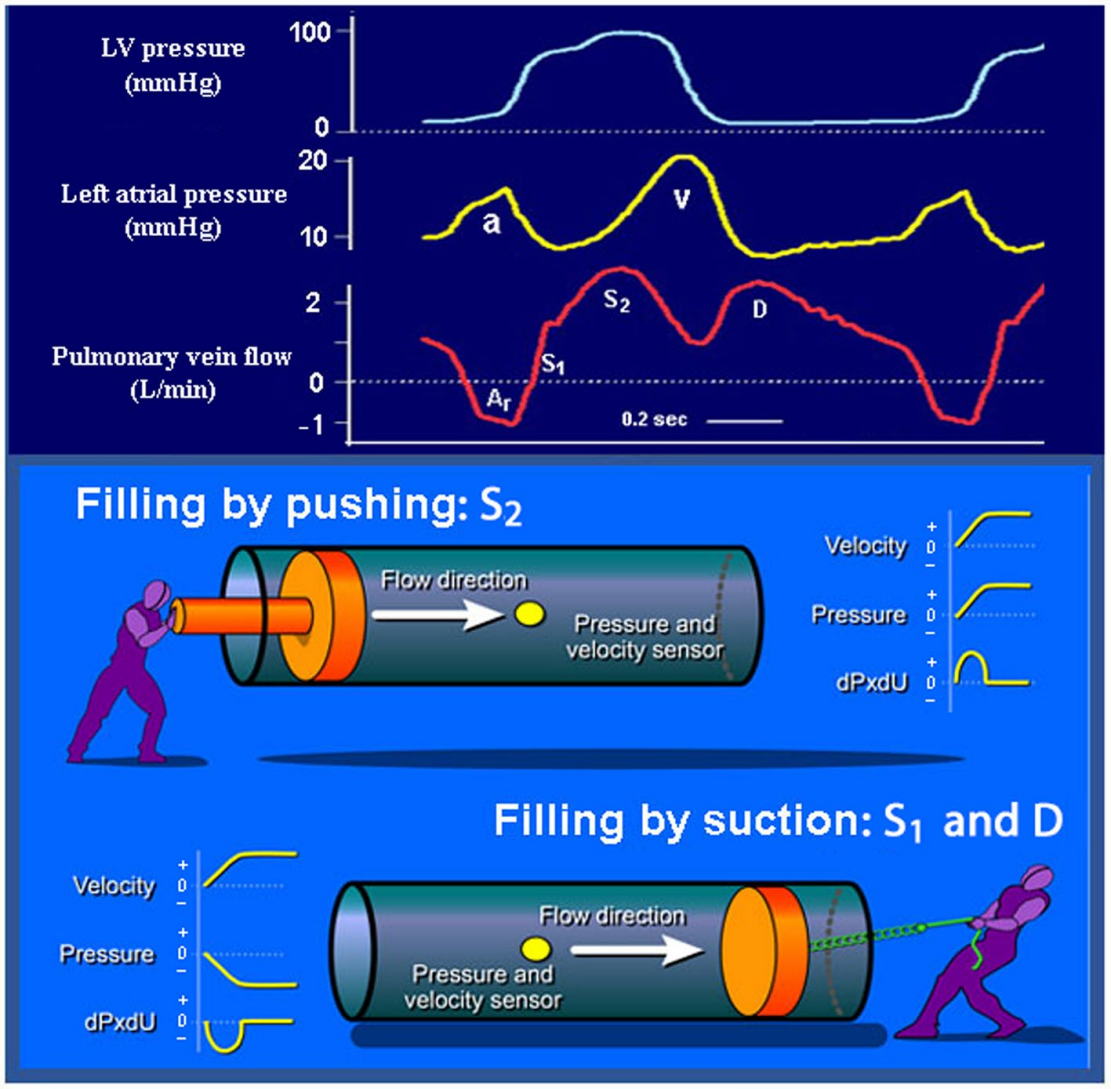
Upper panel: recordings of LV and LA pressures and pulmonary venous flow in a patient prior to coronary artery bypass surgery (same patient as in Fig. 2). Lower panel: *S*_1_ and *D* represent atrial filling by suction caused by fall in LA pressure, and *S*_2_ represents filling caused predominantly by pushing from the RV systolic pressure pulse [6]

The pulmonary venous flow velocity typically has three phases, including a systolic velocity (*S*) with two components, a diastolic velocity (*D*), and reversed flow velocity during atrial contraction (*A*_r_) (Fig. 3). The first component of pulmonary venous systolic flow (*S*_1_) is usually hidden or combined with the second component (*S*_2_), except when there is prolonged atrioventricular conduction or prolonged LV isovolumic contraction time. In a study which utilized wave intensity analysis, it was shown that *S*_1_ is caused by the early systolic fall in LA pressure, and represents atrial filling by suction (Fig. 3) [6]. The magnitude of fall in LA pressure is a function of atrial systolic function and systolic descent of the atrioventricular plane. The *S*_2_, however, is caused predominantly by the RV pressure pulse, which pushes blood forward, and there is a smaller contribution from systolic descent of the atrioventricular plane [6]. Therefore, the magnitude of *S* is determined by RV, LV, and LA systolic function. Furthermore, mitral regurgitation, which is common in heart failure, increases LA pressure and causes reduction of *S* due to lowering of the pressure gradient between pulmonary veins and LA. Therefore, the observation that a reduced *S*/*D* ratio predicts cardiovascular events may be a reflection of reduction in left- or right-sided ventricular function, LA function, and mitral regurgitation, and therefore is a relatively nonspecific marker of cardiac dysfunction.

The pulmonary venous *D*-velocity coincides with the transmitral *E*-velocity, and its magnitude is determined by essentially the same factors that modify mitral *E*. In normal individuals, the pulmonary venous *S* is usually higher than *D*. With advanced LV diastolic function which is characterized by elevated mitral *E*-velocity, there is typically elevated *D* velocity, and for reasons explained in the previous paragraph, there is reduced *S*-velocity. Therefore, the *S*/*D* ratio is usually <1 in patients with heart failure and elevated LV filling pressure. Young healthy subjects with excellent LV relaxation may also have *S*/*D* < 1, but in contrast to patients with advanced diastolic dysfunction, there is not a marked *A*_r_. With increasing LV diastolic pressure, which leads to reduction in operative LV compliance, atrial contraction pushes an increasing volume of blood back into the more compliant pulmonary veins, and this is evident as increase in peak value and duration of *A*_r_ [7, 8].

## Preload and filling pressure

The terms LV preload and LV filling pressure are often used interchangeably when discussing cardiac function, and in most clinical conditions there are concordant changes in the two parameters. Preload refers to how much the myocardium is stretched prior to contraction and is linked to the Frank–Starling law and sarcomere length. The term LV filling pressure refers to the pressure that fills the left ventricle and is used differently depending upon which pressure is available. Both LA mean pressure and LV end-diastolic pressure are used to represent LV filling pressure, but the latter should be preferred when the focus of the study is LV mechanical function. Direct measurement of LA pressure is rarely feasible, but it can be estimated as pulmonary capillary wedge pressure (PCWP) during right heart catheterization and as LV pre-a wave pressure during LV catheterization [9] (Fig. 2).

There are a few important clinical conditions in which LV end-diastolic pressure and left atrial mean pressure do not represent preload. First, in patients on mechanical ventilation and positive end-expiratory pressure (PEEP), there is reduction of LV end-diastolic volume, but elevation of LV end-diastolic pressure due to increase in extracardiac pressure (pericardial pressure). In these patients, LV preload can be measured as transmural end-diastolic pressure, which is the effective filling pressure. Since pericardial pressure can be approximated as mean right atrial pressure [10], LV transmural filling pressure during PEEP can be calculated as PCWP minus mean right atrial pressure [11]. Another example of divergence between LV end-diastolic pressure and preload is in heart failure patients receiving intravenous vasodilator therapy. Then there may be marked acute reduction in LV diastolic pressures with little or no change in end-diastolic volume, and the reduction in LV end-diastolic pressure has been ascribed to reduction in pericardial pressure [12].

## Relaxation, restoring forces, and diastolic compliance

**Fig. 4:**
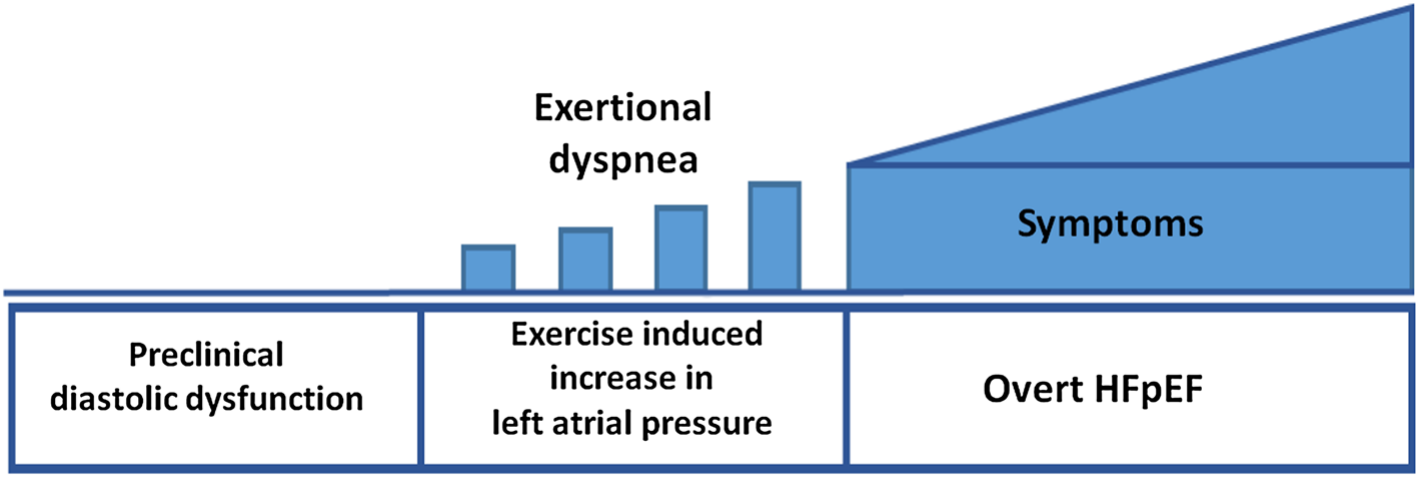
Schematic illustration of progression from preclinical diastolic dysfunction to heart failure. Modified from Ohara et al. [28]

The fundamental mechanisms of diastolic dysfunction are impaired relaxation, loss of restoring forces, and increased diastolic stiffness, and as a compensatory mechanism to maintain cardiac output, there is elevation of LA pressure. The latter response involves reflex mediated venoconstriction with translocation of blood towards the central circulation, which is a fast response, and fluid retention in the kidneys as a slower mechanism. In the early phase of diastolic dysfunction, LV filling pressure may be normal at rest and elevated only during physical exercise (Fig. 4).

Typical for normal diastolic function is rapid relaxation and vigorous restoring forces which result in low or even negative minimum LV diastolic pressure, causing high mitral pressure gradient and dominance of early diastolic LV filling. Myocardial relaxation reflects rate of calcium reuptake from the cytosol. Restoring forces reflect LV systolic function and are generated when the ventricle contracts below its unstressed volume, analogous to manual compression of a tennis ball which recoils back to its original round shape when compression is released. When there is normal rapid LV relaxation, restoring forces generate negative minimum diastolic pressures and imply that the LV wall performs work to pull blood into the ventricle, representing filling by suction. There is also an alternative definition of suction, which is LV filling during falling pressure, which does not include negative LV pressure or release of restoring forces. This definition also is useful, as shown in studies of LA filling, as illustrated in Fig. 3.

**Fig. 5:**
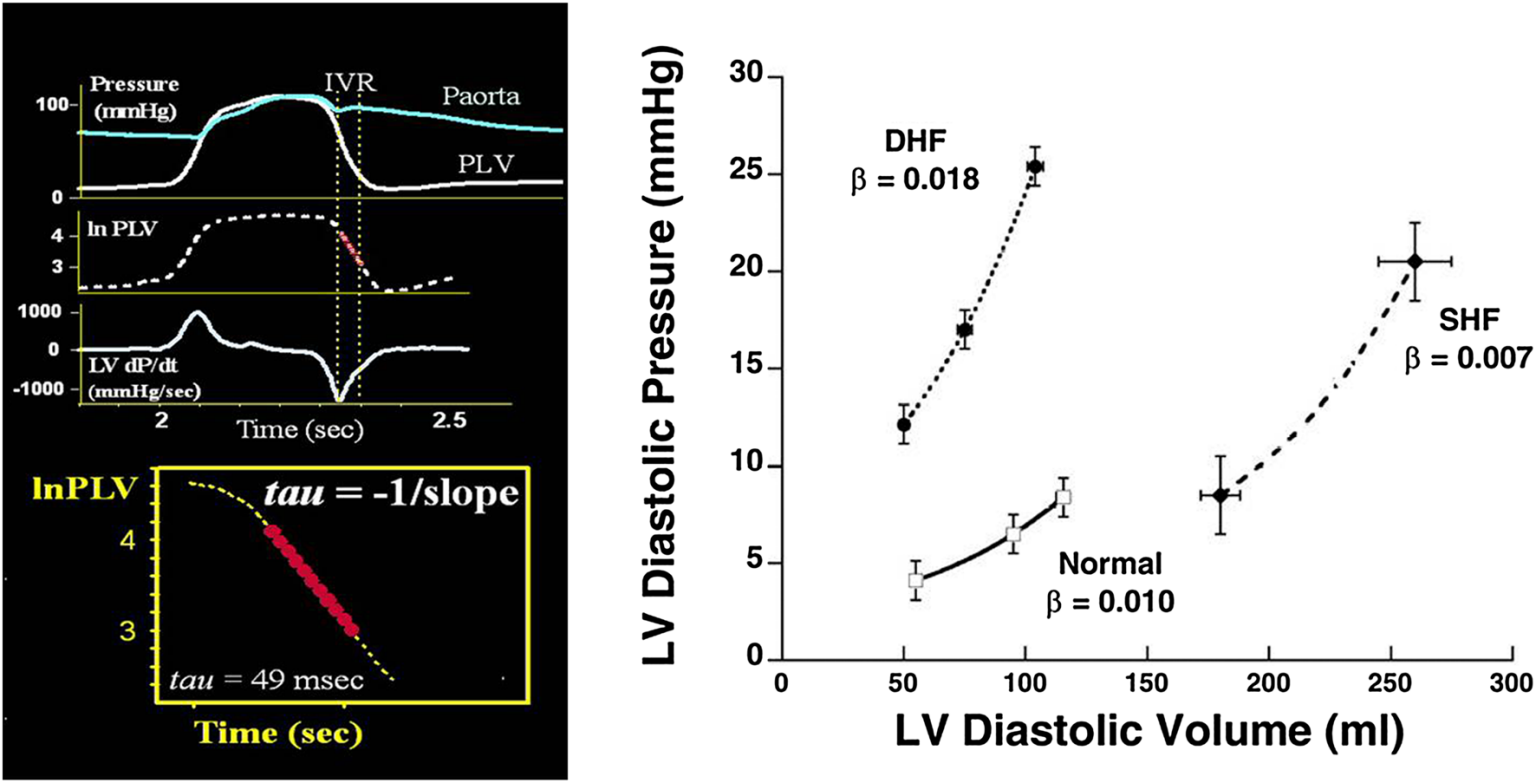
Invasive measures of diastolic function: Left panel shows calculation of the time constant of LV isovolumic pressure fall (*tau*). Recordings from Smiseth et al. [29]. Right panel shows LV diastolic pressure–volume relations from normal controls (solid line) and patients with normal EF, diastolic heart failure (DHF) (dotted line), and reduced EF, systolic heart failure (SHF) (dashed line) [30]

Relaxation and restoring forces may be evaluated by measuring *e*′ and untwisting velocity by echocardiography [13–16], but currently only *e*′ is used clinically, as there are methodological challenges with measuring untwisting velocity. When invasive pressure is available, the time constant of LV isovolumic pressure fall may be used to quantify LV relaxation [17], but currently mainly for research purposes (Fig. 5). Relaxation and restoring forces exert their effects simultaneously, and clinically there is no way to measure each component separately.

**Fig. 6:**
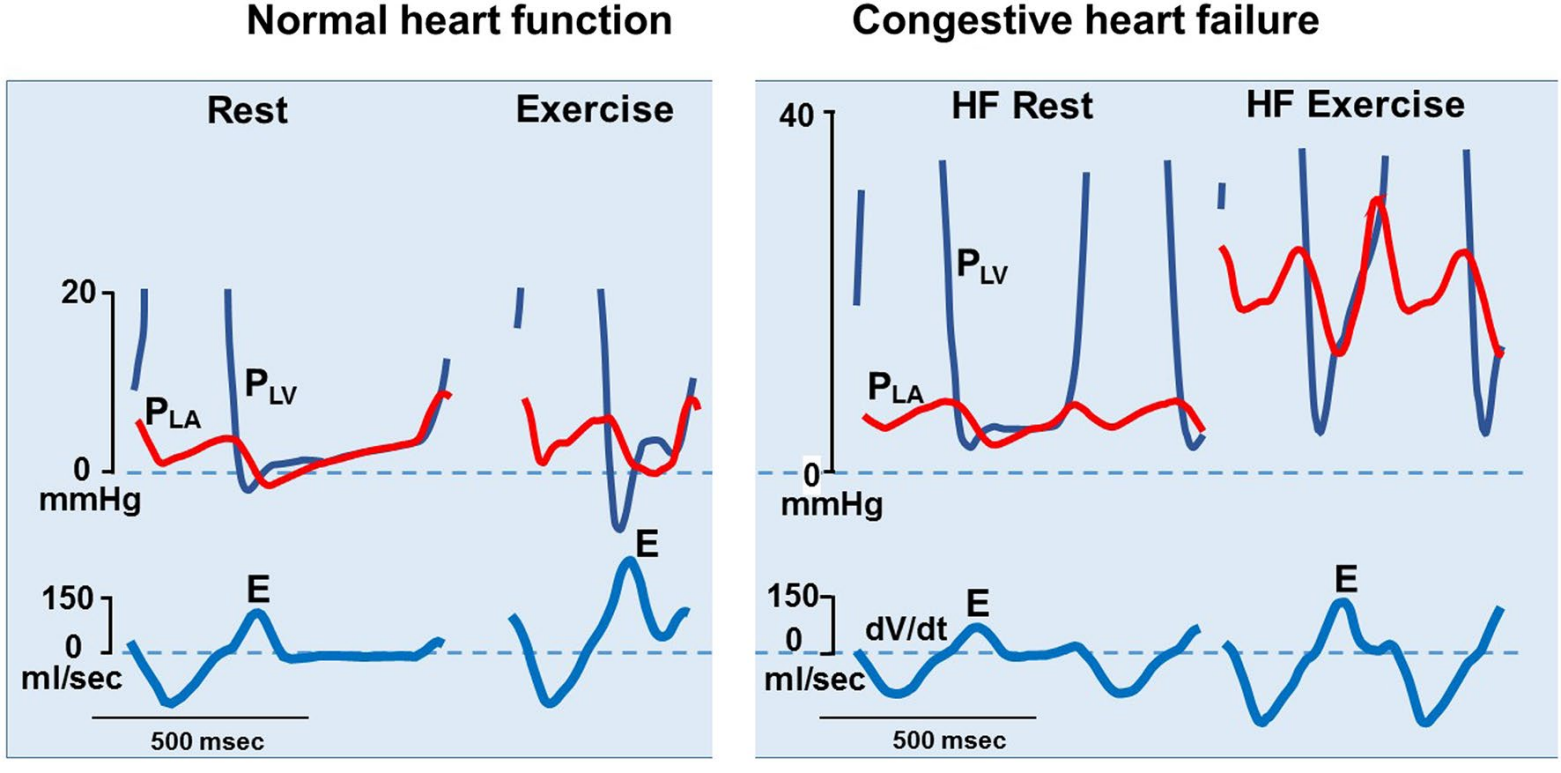
Loss of diastolic suction in the failing heart: Experimental study showing a normal heart (left panel), which generates markedly negative diastolic pressure during exercise, causing LV filling by suction. Thereby, the normal heart can increase mitral *E* with no rise in left atrial (LA) pressure. During heart failure (right panel), LV minimum pressure does not decrease during exercise and transmitral flow increases by elevation of LA pressure. Transmitral flow rate was measured as d*V*/d*t* [18]

Experimental data show that the normal LV may generate negative early diastolic pressure which sucks blood into the ventricle. This mechanism is attenuated or lost in heart failure, as demonstrated by Little’s group in chronic dog experiments (Fig. 6) [18]. Presumably, a similar mechanism is operative in normal human hearts and causes filling by suction [19, 20]. The interaction between LV end-systolic volume, restoring forces, and diastolic filling illustrates the tight coupling between systolic and diastolic function. Mitral-to-apical flow propagation velocity by color flow imaging is a parameter of early diastolic function, but is currently not widely used [21].

Left ventricular diastolic compliance is a lumped parameter and is determined not only by LV myocardial compliance, but also by elastic properties of extraventricular structures (pericardium and lungs) and by right ventricular diastolic pressure. The term LV chamber compliance is often used rather than just LV compliance, with the latter referring to myocardial compliance. Because the LV pressure–volume relationship is curvilinear, chamber compliance is a function of the operative LV diastolic pressure. As an example, elevation of LV diastolic pressure by volume loading, which moves the LV pressure–volume coordinate to a steeper part of the pressure–volume curve, causes reduction in chamber compliance in a ventricle which is entirely normal. Therefore, reduced chamber compliance does not necessarily mean there has been a change in myocardial elastic properties. Figure 5 shows diastolic pressure–volume curves from a group of normal individuals compared with patients with heart failure and preserved EF and reduced EF, respectively.

**Fig. 7:**
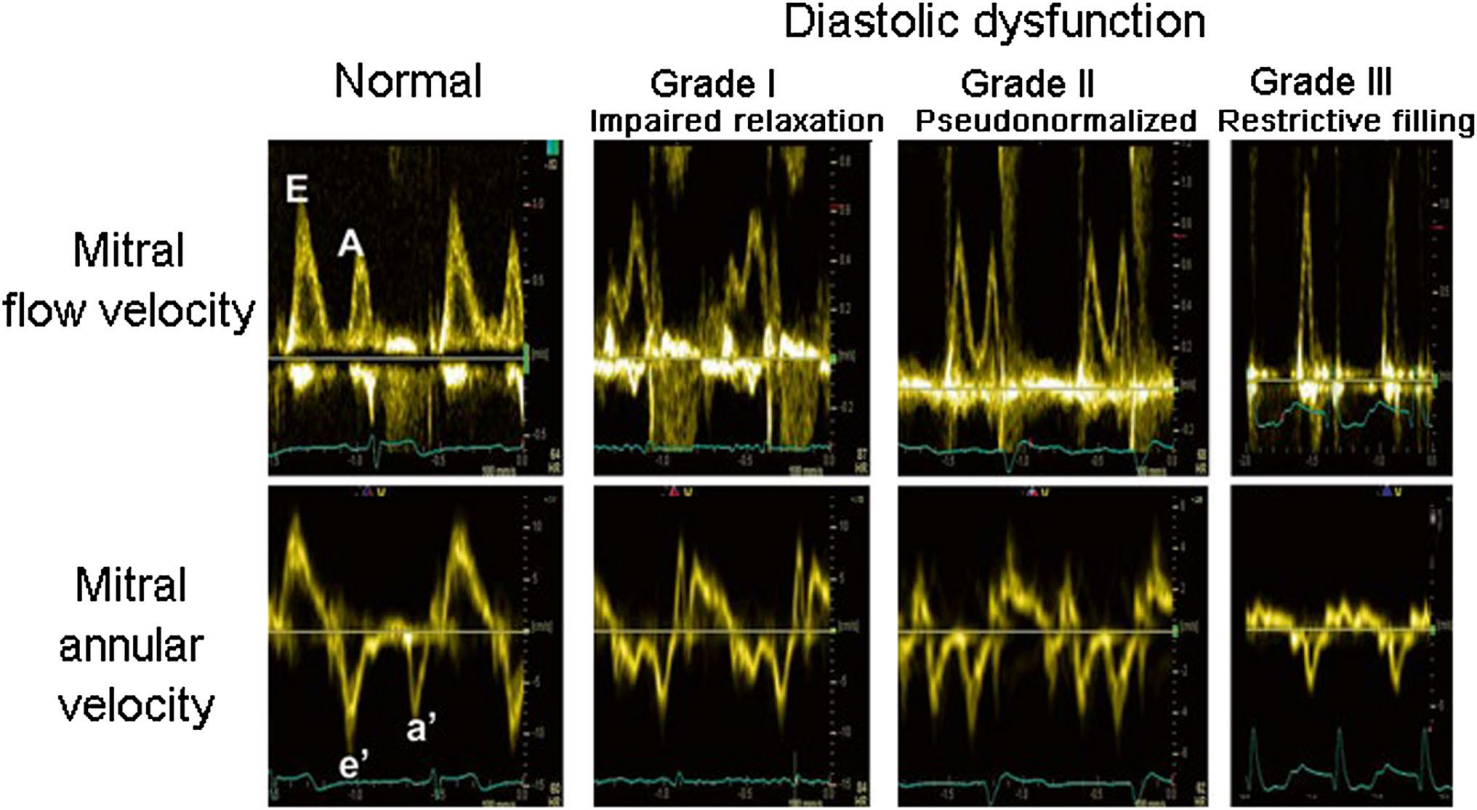
Mitral filling patterns (upper panels) and mitral annular velocities (lower panels) in subjects with different grades of LV diastolic dysfunction

**Fig. 8:**
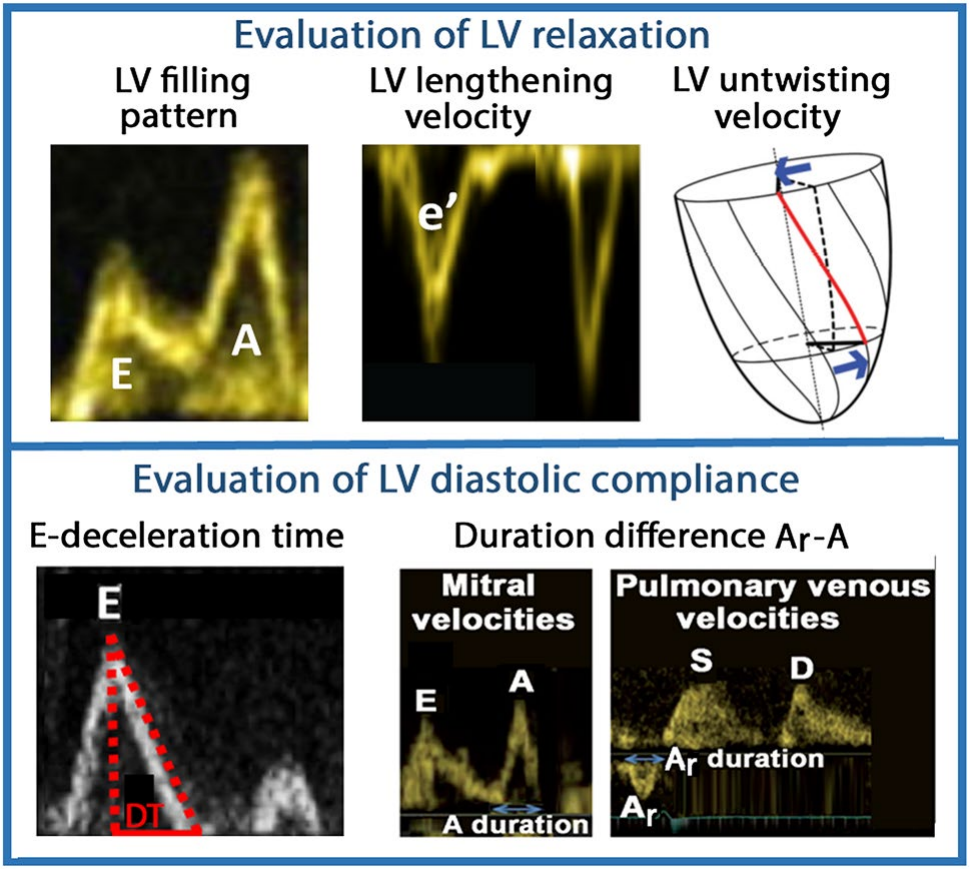
Noninvasive methods to evaluate LV relaxation and diastolic compliance

**Fig. 9:**
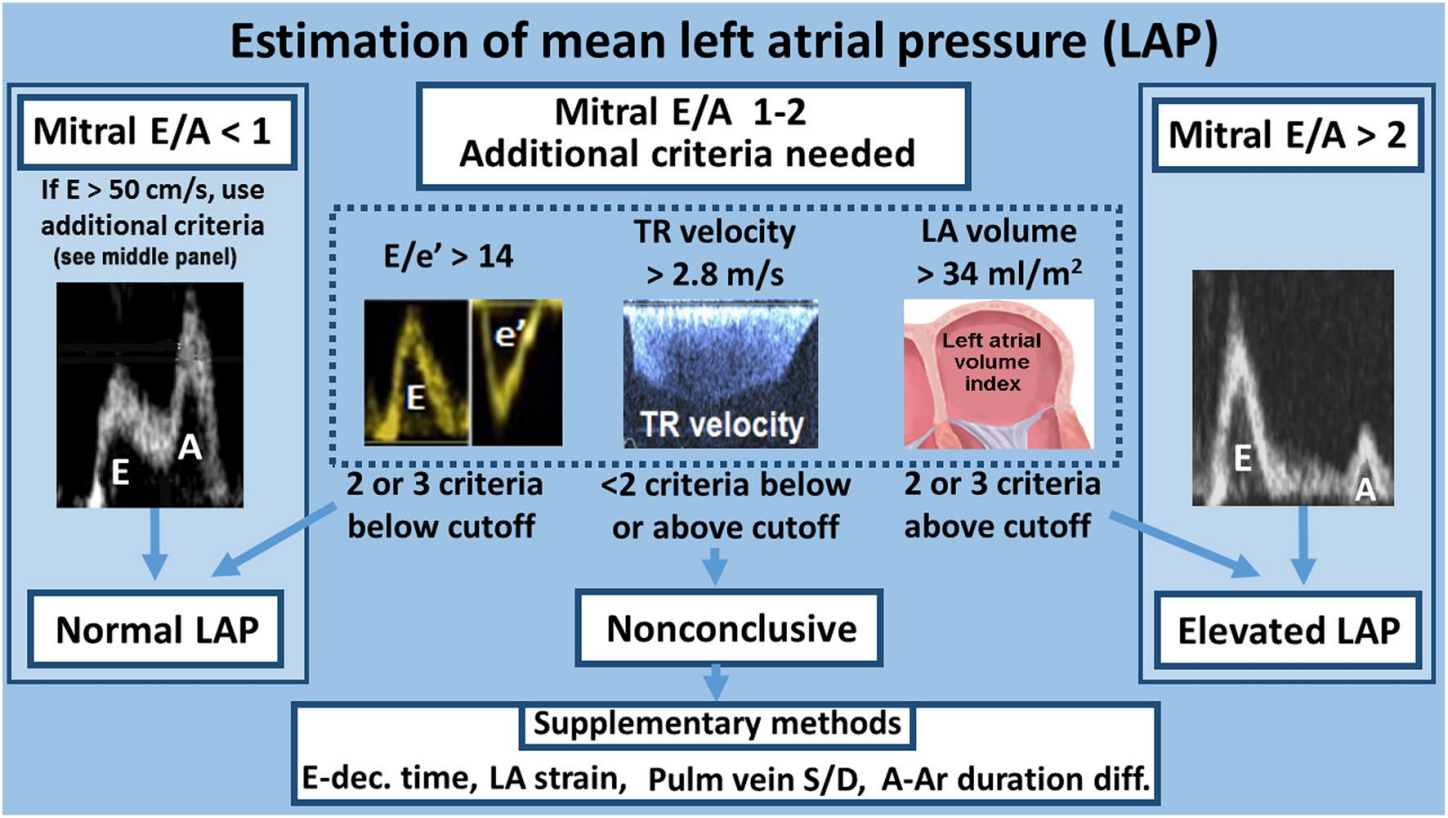
Assessment of LV filling pressure by echocardiography according to ASE/EACVI recommendations [1]

Different mitral filling patterns are displayed in Fig. 7. The pattern of impaired relaxation (grade I) has low *E* and tall *A*. The pattern of pseudonormalized filling (grade II) has flow velocities similar to a normal heart and is identified by reduced *e*′. The pattern with tall *E* with short deceleration time (<150 ms), combined with small *A* and *E*/*A* > 2 is named restrictive filling (grade III) and is associated with reduced LV diastolic compliance [22] (Fig. 8). By definition, grades II and III diastolic dysfunction have elevated LV filling pressure, which can be determined according to the algorithm presented in Fig. 9. The relationship between *E* deceleration time and degree of diastolic dysfunction is nonlinear; therefore, in mild diastolic dysfunction dominated by impaired relaxation, there is prolongation of *E* deceleration time because of ongoing relaxation during flow deceleration. Importantly, in young healthy individuals with high mitral *E*, there is often a deceleration time <150 ms. Therefore, a short deceleration time should not be used as a standalone index of reduced LV compliance. Healthy people, however, do not have a large *A*_r_, which is typical for ventricles with reduced compliance as discussed below.

Reduction in LV chamber compliance is also reflected in attenuation and abbreviation of the transmitral *A* velocity and is typically combined with accentuation and prolongation of the pulmonary vein *A*_r_ [7, 8]. When the atrium contracts against a ventricle with reduced compliance, little blood moves forward across the mitral valve, antegrade mitral flow is interrupted prematurely, and instead blood regurgitates into the more compliant pulmonary veins. When the duration of *A*_r_ exceeds the duration of mitral *A* by >30 ms, it is consistent with elevated LV end-diastolic pressure. The limitations of *A* duration difference as an index of diastolic compliance include atrial mechanical failure and suboptimal quality of the pulmonary venous flow signal. Furthermore, even in patients with severe diastolic dysfunction, a large *A*_r_ may be absent when there is prolonged atrioventricular conduction or tachycardia, so that atrial contraction takes place before diastolic pulmonary venous flow (*D*) is completed. In these cases, the difference in *A* duration does not reflect LV filling pressure.

Thus, a short mitral *E* deceleration time together with a small and abbreviated mitral *A* and a large *A*_r_ of long duration are consistent with reduced LV compliance.

## Left atrial volume and strain

Whereas Doppler-derived diastolic indices reflect LV filling pressures at the time of measurement, LA volume reflects the cumulative (chronic) effect of LV filling pressures over time in patients who are in sinus rhythm and do not have mitral disease, anemia, or other high-output states. LA volume >34 mL/m^2^ is considered enlarged [1]. There is also an overlap of LA volume between healthy individuals and subjects with diastolic dysfunction, and LA volume can be increased in elite athletes.

More recently, global LA strain by two-dimensional (2D) speckle tracking echocardiography was introduced as a promising supplementary marker of LV filling pressure. Elevated LA pressure is associated with low values for LA reservoir strain. The incremental value of measuring LA strain remains to be defined, but preliminary data from smaller studies are promising.

## Left ventricular geometry and strain

In patients with heart failure symptoms and normal LVEF, the finding of LV hypertrophy favors HFpEF as diagnosis. After introduction of strain imaging by speckle tracking echocardiography, it became apparent that patients with normal LVEF may have mildly reduced LV systolic function by global longitudinal strain. Therefore, LV strain imaging represents a supplementary test which is useful when echocardiographic indices of diastolic function are inconclusive and it is not clear whether the patient suffers from heart disease. Reduced global longitudinal strain provides support in favor of heart disease as underlying mechanism of symptoms.

Diastolic dysfunction is present in arterial hypertension, diabetes mellitus, obesity, and in a large number of cardiovascular disorders often at a preclinical stage, and is part of normal aging. Diastolic dysfunction may be graded as illustrated in Fig. 7. Since transition from normal function to diastolic dysfunction is gradual, it is not obvious what should be used be used as criteria for diastolic dysfunction. In the recent American Society of Echocardiography/European Association of Cardiovascular Imaging (ASE/EACVI) recommendations, diastolic dysfunction was considered present when more than 50 % of the parameters *e*′, *E*/*e*′, LA volume index, and peak tricuspid regurgitation velocity were positive [1]. This relatively strict definition was chosen to make the criteria more specific than in previous guidelines, but implies reduced sensitivity. Importantly, this recommendation is based on expert opinion and has not been validated against invasive reference methods for diastolic dysfunction.

When the purpose of the study is to determine whether a patient has elevated LV filling pressure, the ASE/EACVI guideline recommends using the algorithm presented schematically in Fig. 9. The rationale behind the conclusion that LA pressure is normal when mitral *E* is <50 cm/s combined with *E*/*A* < 1 is that a low *E* implies a small transmitral pressure gradient. The combination with *E*/*A* < 1 confirms that *E* is low also when compared with *A*. When *E* is tall and much higher than *A*, it implies a high transmitral gradient, which in turn implies high LA pressure. One exception is young healthy individuals, who may have negative early diastolic pressure and therefore high gradient and tall *E* with normal LA pressure. For intermediate mitral filling patterns, the recommendation is to use *E*/*e*′, LA volume, and peak TR velocity in combination (Fig. 9). The reason why elevated *E*/*e*′ is useful is because combination of high transmitral gradient (high *E*) on top of elevated minimum LV diastolic pressure (suggested by low *e*′) means high LA pressure. Large LA reflects long-term effect of elevated LA pressure. High TR regurgitation velocity implies high pulmonary artery systolic pressure. It was recently confirmed in two large multicenter studies that this algorithm can identify patients with elevated filling pressure with high feasibility and good accuracy [2, 3].

A common misinterpretation of the ASE/EACVI guideline [1] is that evaluation of filling pressure should start with a diagnostic algorithm to determine whether the patient has diastolic dysfunction or not. This is not needed when there is clinical suspicion of heart disease; Then one can go directly to the algorithm in Fig. 9. In a similar way as in HFpEF, the algorithm in Fig. 9 can be used to assess filling pressure in patients with reduced EF [1, 2].

When evaluating patients with potential HFpEF, one should always search to exclude diseases such as valve disease, coronary artery disease, pericardial disease, and right-sided heart disease before it is concluded that a patient suffers from HFpEF. Furthermore, since LV diastolic pressure may be entirely normal at rest in HFpEF, a noninvasive and sometimes invasive diastolic stress test is recommended when there is inconsistency between symptoms of heart failure and echocardiographic findings at rest [23, 24]. Importantly, one should consider pericardial disease as underlying disorder [25].

## Pulmonary artery pressure

When pulmonary hypertension is observed in combination with signs of LV disease, it is consistent with elevated LV filling pressure. Pulmonary artery systolic pressure can be estimated in most patients by continuous wave Doppler of tricuspid regurgitation velocity and an estimate of right atrial pressure.

When the tricuspid velocity cannot be imaged or the peak of the velocity is not well defined, pulmonary artery acceleration time may be used as an index of elevated pulmonary artery pressure and is measured with high feasibility as the time interval between the onset and peak pulmonary arterial systolic flow velocity [26]. Values <100 ms are consistent with elevated mean pulmonary artery pressure.

## Key points

Left ventricular diastolic function can be evaluated by measuring indices of LV relaxation, restoring forces, diastolic compliance, and filling pressure. By using a combination of indices, diastolic function can be graded and LV filling pressure estimated with high feasibility and good accuracy regardless of level of systolic function. Evaluation of diastolic function should be considered in the following clinical settings:

1. In patients with unexplained exertional dyspnea or other symptoms or signs of heart failure which cannot be attributed to reduced LV systolic function or to diseases such as coronary artery disease, valve disease, pulmonary vascular disease, lung disease or other diseases.
2. To determine if LV filling pressure is elevated in patients with heart failure and reduced LV ejection fraction when considering adjustment of diuretics or other heart failure medication.
3. Grading of diastolic function can be used for risk assessment in asymptomatic subjects and in patients with heart disease.
